# Experiences of university teachers with rotational blended learning during the COVID-19 pandemic: A qualitative case study

**DOI:** 10.1371/journal.pone.0292796

**Published:** 2023-10-12

**Authors:** Ahmed Alkaabi, Ahmad Qablan, Fatima Alkatheeri, Aisha Alnaqbi, Maha Alawlaki, Latifa Alameri, Bushra Malhem

**Affiliations:** 1 Department of Foundation of Education, College of Education, United Arab Emirates University, Al Ain, United Arab Emirates; 2 Department of Curriculum and Instruction, College of Education, United Arab Emirates University, Al Ain, United Arab Emirates; Satyawati College (Eve.), University of Delhi, INDIA

## Abstract

This qualitative case study examines the self-efficacy of university teachers during the COVID-19 pandemic as they struggle to incorporate new technology, teaching strategies, and curriculum delivery in the shift from total remote learning to biweekly rotation learning––two weeks of face-to-face learning and two weeks online. This study was conducted over one full semester among university teachers teaching undergraduate students with the rotation model at one federal university located in the United Arab Emirates. A case study design was used as a methodology to guide this research with a primary data collection method of semi-structured interviews of 11 teachers corroborated by both in-person and online classroom observations. Participating teachers were from various colleges within the university, including medicine, education, business, law, humanities, and science. The data from the interviews and observations were analyzed using thematic analysis, which yielded the following six themes: (1) continuously changing expectations, (2) mixed feelings regarding technology self-efficacy, (3) loss of learning among undergraduate students, (4) trial and error with teaching strategies, (5) the need to consult with students in the teaching and learning process, and (6) the shift from struggle to resilience. The results of the study indicated that having clearer expectations, proper technology training, and intradepartmental collaboration may help educators overcome the challenges associated with the hybrid rotation model. These results are expounded thoroughly along with relevant implications for robust leadership practices to enhance the quality of teaching and learning during potential future crises.

## Introduction

The beginning of 2020 marked an unforgettable, critical point within the education memory threshold, which led to unprecedented changes within the global sectors [1–3] particularly in the educational system. During the COVID-19 outbreak, Emergency remote teaching (ERT) turned the landscape of teaching and learning upside down and pushed it into uncharted territory. In fact, the COVID-19 pandemic is the first global health crisis to transpire in the digital age [4, 5], and it has significantly impacted both students [6, 7] and teachers at all levels of education. Teachers all over the world have had to quickly adjust to remote learning by adopting new teaching methods and becoming familiar with various new technologies and applications. Unfortunately, however, even in the best-adjusted schools, ERT remains a difficult and challenging experience for both instructors and learners [8].

With the uncertainties created by the COVID-19 pandemic, universities and schools had to devise different strategies to maintain the continuity of education, while ensuring the safety of students and teachers [9–11]. Schools began implementing remote education with the usage of online tools (e.g., Teams, Zoom, Blackboard, etc.) to induce some level of learning stability. Later, when the situation improved, universities and schools gradually returned to a face-to-face modality of learning. However, they could not expect a complete return to traditional on-site learning due to the uncertainty of the epidemiological circumstances [12]. COVID-19 has been tricky and confounding to scientists as it has evolved, and each variant has had different effects, making it harder to predict [13]. Given that the pandemic continues to affect education, employing a flexible mode of teaching and learning has become the focal point of many educational institutions. Blended learning or hybrid learning has grown massively in popularity, and the transition to hybrid learning has transpired rapidly. Although a growing body of literature has been dedicated to unravelling the challenges of schoolteachers with hybrid learning, little has been done in the area of higher education to examine the self-efficacy of university teachers (i.e., instructors, assistant professors, associate professors, professors) as they adapt to it. For example, there is a dearth of literature on how university teachers cope with required changes in the use of new technologies, curriculum delivery, and instructional strategies [14]. Although virtual and hybrid instructional models are discussed in the literature as complements to traditional instructional practices, [15] pointed out that there was a paucity of literature investigating teachers’ perceptions of the sudden shifts from one model to the other during emergencies.

University teachers encounter frequent setbacks when implementing the hybrid learning system because they are not accustomed to its various facets [16]. Researchers need to capture and understand these challenges that faculty members face. This study will emphasize the need to improve the self-efficacy of teachers as they battle with the changes brought about by hybrid learning. The overriding question of this study is, how do university teachers experience changing self-efficacy as they grapple with teaching undergraduate students in hybrid learning environments, in terms of the use of technologies, delivering curriculum, and adopting new teaching strategies? This qualitative case study will be conducted among undergraduate students at one federal university located in the United Arab Emirates. The university was the first in the area to employ the rotational model of blended learning. A group of administrators were tasked with reopening the university to students. They accomplished this by splitting the students into two groups, one of which would attend classes in person while the other would attend online during the same time period. All the while, the taskforce closely followed the instructions and guidelines set forth by the health authorities regarding COVID-19. Every two weeks, those students who attended classes in person were assigned to attend class online for the next two weeks, and those who had attended class online before were assigned to attend in person.

This biweekly rotation continued throughout the remainder of the Fall Semester 2021, always in keeping with the health directives of the authorities overseeing the pandemic to allow learning to continue while also keeping students, teachers, and administrators protected and safe. As part of the new protocols, adjustments were required by stakeholders at every level, which caused no insignificant amount of strain on all involved. Perhaps more than any other group, university teachers were weighed down with enormous burdens associated with countless unprecedented requirements and changes to their usual patterns of instruction. Researching this group is necessary to uncover how university teachers experienced the emergency changes, particularly with regard to their self-efficacy as it pertains the various aspects of their jobs as instructors of undergraduate students. This research has empirical, theoretical, and practical significance for faculty members and administrators working at the university level. A deeper understanding of how teachers respond to the transition to hybrid learning may help university officials provide better services needed for their personnel. Additionally, understanding the challenges experienced by faculty members may better prepare educators to face similar emergent situations.

## Literature review

This study focuses on university lecturers, who had to fulfill the difficult task of converting their teaching materials into new online modules. Although the rapid transition to ERT allowed for the continuity of universities’ instruction, preparing high-quality online materials would require more time and effort from university lecturers to prepare workable online learning modules [17, 18].

However, in the new ERT reality, university lecturers experienced additional demands as they suddenly had to incorporate academic, technical, guidance, social, and organizational functions related to online teaching. Thus, a new set of skills (i.e., pedagogical, cognitive, technological, communicative, and personal) was needed to help them deliver quality online teaching for their students [19]. Generally, studies showed that university lecturers were able to accommodate that demand by utilizing their previous knowledge of technology [20, 21]. However, some other lecturers experienced diverse personal and physical burdens to switch to ERT [17]. Examples of personal factors that facilitated the transition to ERT were; their positive attitude toward utilizing digital technologies in their teaching [22, 23] as well as their strong self-efficacy expectancy for online teaching [24].

### The significance of self-efficacy

In his theory, [25] elucidated that the more teachers believe in themselves and their abilities, the more likely they will be able to help their students make noticeable learning progress. Applying this theory to the context of ERT, we find that the more teachers understand hybrid learning, the better the chance for student prosperity within that learning modality. In battling the inescapable challenges that arise from ERT, university teachers can increase and improve their self-efficacy to improve outcomes [26]. [12] defined self-efficacy as (1) the belief in one’s ability to accomplish a goal and (2) the estimation of how likely one’s behavior will lead to desired results [27]. [28] defined it as a "perception of a person’s ability to organize and execute specific courses of action required to accomplish a specified objective" (p. 391). Therefore, it can be inferred that self-efficacy may be altered by changing skill levels or from situation to situation [29].

### The relationship between self-efficacy and technology, curriculum, and teaching

Technology self-efficacy measures an individual’s ability to successfully complete tasks using technological tools [30]. It assesses how effectively a teacher integrates technology into the classroom to enhance student learning [31]. Self-efficacy directly influences behavior, making it crucial to understand teachers’ positions regarding technology use in education. A study by [32] found that educators with prior online teaching experience reported high self-efficacy levels, while those without such experience faced challenges. Teachers often seek assistance from colleagues and administrators to navigate classroom technology and may need encouragement to incorporate technological tools into their teaching methods [33–36].

Advocates for technology integration in education argue that technology self-efficacy is not the only factor determining successful classroom outcomes. They contend that while technological proficiency is valuable, it should not overshadow other essential teaching skills and pedagogical strategies [37]. Overemphasis on technology self-efficacy can divert focus from broader educational goals. Even tech-savvy teachers may struggle to integrate elements like formative assessment and collaboration into their hybrid classrooms [38–40]. These challenges directly affect teachers’ self-efficacy, which, although difficult to quantify precisely, can often be assessed by examining teachers’ challenges, readiness, and achievements [41, 42]. [43] emphasized the importance of assessing teachers’ readiness for successful technology integration. [44] suggested that effective incorporation of ICT resources requires proficiency in both pedagogical and technological domains.

As far as curriculum, [45] noted that online instruction is more challenging than face-to-face teaching because teachers must manage and interact with students in a virtual setting. Planning online lessons is also more complex and time-consuming. However, research by [46] indicated that blended learning outperforms traditional education. Blended learning offers numerous benefits, such as access to resources, collaboration, flexibility, and learner motivation [47]. Effective distance learning involves various factors, including curriculum accessibility, learner experience, and student support [48]. Teachers with higher self-efficacy experience less stress during curriculum changes but may become less confident under high pressure [49]. Teachers must continually update their content knowledge to maintain high self-efficacy levels [50].

Self-efficacy is a critical motivator affecting teachers’ attitudes and actions [51]. Research shows a positive correlation between teachers’ self-efficacy and their enthusiasm for teaching [52]. Teachers with higher self-efficacy levels are more likely to exhibit quality teaching, vary instructional practices, and create engaging lesson content [53–58]. Given the drastic changes in instructional environments due to COVID-19, it is imperative to consider multiple factors that influence teachers’ self-efficacy, such as confidence in using technology, available infrastructure [59], and institutional support [60]. This study aims to fill a research gap by exploring the impact of shifting from total remote learning to biweekly rotation learning on university lecturers’ self-efficacy.

## Materials and methods

The review of related studies showed that the majority of research on teachers’ self-efficacy was either quantitative experimental or quantitative correlational. However, these tools might not be suited for studying a phenomenon such as self-efficacy as it is complex and context-specific. Interpretative tools like the case study, ethnography, and grounded theory might uncover a clearer representation of the concept. This study will employ a qualitative research approach in line with the purpose of the study. According to [61], qualitative research is used to collect a detailed account of human behavior and beliefs within the contexts in which they occur. [62] emphasized that qualitative research involves an interpretive and naturalistic approach that looks through the lens of “things in their natural settings” in an attempt to “make sense of, or to interpret, phenomena in terms of the meanings people bring to them” (p. 3). This study will examine participants’ perceptions and interpretations of how university teachers experience changing self-efficacy as they grapple with teaching undergraduate students in hybrid learning environments, in terms of the use of technologies, delivering curriculum, and adopting new teaching strategies. A case study design will be used as a methodology embedded within this qualitative research study. As [63] clarified, the popularity of the case study design stems from its ability to provide the researcher with a deeper understanding of specific individuals, an identified problem, or a distinctive situation by closely studying the phenomenon in intensive and great depth. Another merit of the case study design is that it enables the researcher to triangulate the collected data through interviews, observation, and documents. Data triangulation establishes credibility by examining data from multiple perspectives with the intention of finding consistencies across the data sources. The primary data collection methods utilized in this study are semi-structured interviews of 11 university teachers and observations across online and face-to-face sessions. Data were collected from June 2022 until September 2022 and then researchers started to analyze the collected data during the Fall semester of 2022.

### Recruitment and sample size

Participants in this study were recruited using purposeful sampling methods [64]. Selecting appropriate individuals with knowledge and experience about the topic of interest was necessary to ensure a successful sample [63]. [65] identified several important elements to consider while implementing purposeful sampling, the foremost of which were the interest and ability of the participants. He further expounded the significance of participants’ ability to reflect inwardly and express their experiences and views. In this instance, the purposeful sampling was guided by specific criteria, described by [64] as ‘criterion sampling’ or a checklist of criteria used to select participants. Each participant underwent screening and was required to meet three criteria: (1) actively working as an undergraduate teacher; (2) participating in the rotation modality of blended learning––alternating between two weeks in person and two weeks online; and (3) being open to discussing their experiences.

The ideal sample quantity for a case study is not universally agreed upon [66]; however, for this study, 11 university teachers were recruited to participate. Five of them were male, and six were female. Each participant was given a consent form to acknowledge their agreement to participate in the study. Participants were assured that their participation is completely optional and they can withdraw from participating at any time. Additionally, the authors confirmed to participants that their personal information will not be collected or identified by any means and their responses will solely be used for this study only.

In order to gather details, locate data redundancies, and achieve data saturation, the researchers held two-hour interviews with each participant. Table 1 displays each teacher’s gender, title, university affiliation, and years of experience.

**Table 1:** Summary of university teachers––names are pseudonyms.

Name	Gender	Title	Experience (# Years)	College
Anwar	Male	Instructor	4	Education
Saif	Male	Associate Professor	7	Humanities and Social Sciences
Yousef	Male	Assistant Professor	17	Education
Michel	Male	Associate Professor	15	Medicine and Health Sciences
Sami	Male	Assistant Professor	10	Information Technology
Amna	Female	Associate Professor	19	Education
Diana	Female	Associate Professor	11	Humanities and Social Sciences
Hessa	Female	Associate Professor	14	Law
Maha	Female	Instructor	13	Information Technology
Reem	Female	Associate Professor	7	Science
Lexi	Female	Assistant Professor	9	Education

### Interviews

The primary data collection method used in this study was interviewing, which was used to obtain detailed data on the teaching practices within the university during the emergency implementation of the rotational model of blended learning. The interviews were conducted with the central purpose of making sense of the participants’ experiences, as opposed to focusing on testing a particular hypothesis, in keeping with [67] recommendation. The researchers interviewed the participants for two hours in person to understand their perspectives on their experiences with the rotational learning model during the 2021–22 academic year. The interviewer followed a semi-structured style of questioning in order to maintain enough flexibility and allow for a smooth and open conversation. As [64] explained, interviewing in a semi-structured manner allows the researcher to insert additional questions or change existing ones that might be better suited for this study. The semi-structured interviews followed five guiding rules: (1) questions that introduced participants to the topic being studied, (2) open-ended questions that were conducive to a comfortable discussion about the research while still focusing on a particular topic, (3) questions that allowed the participating teachers to include any additional comments or information that may not have been covered, (4) a short introduction before each segment of the interview, and (5) an expression of gratitude at the end of the interview for the time and participation of each of the teachers [68].

### Observations

In order to add depth to the data and strengthen the results of the study, the researchers conducted direct observations. This approach enabled the researchers to passively examine the pedagogical practices of the teachers with a complete focus on documenting occurrences within the classroom. Specifically, this non-participant observation technique allowed the investigators to witness the phenomena transpiring organically from a distance without interfering with the activities taking place. The researchers situated themselves in the classroom setting, either virtually or in-person, and meticulously recorded all observable incidents related to the study objectives. This separation from the activities in the classroom allowed the observer to come to a deeper understanding of the phenomenon under study, as [64] suggested.

A total of 22 observations were conducted, with each university teacher being observed twice across six distinct colleges: Education, Humanities and Social Sciences, Medicine and Health Sciences, Information Technology, Law, and Science. The researchers conducted each observation in 60-minute segments, taking open-ended field notes throughout each session with as much detail as possible about the teachers’ self-efficacy, new teaching strategies, and the use of technology. The observations were conducted during both in-person and online classroom sessions, and observations continued until data saturation was reached. [69] suggested that data saturation can take anywhere from days to several years depending on the phenomenon being studied. Due to the nature of the present study, data saturation was fully reached after several months.

### Data analysis

The data analysis was conducted using a thematic approach, in adherence with [70] six-phase process. Insights from [71] were also incorporated, particularly in the selection of recurring themes within the interview transcripts and field notes. The first phase involved becoming familiar with the data, where the researchers engaged in multiple readings of the interview transcripts and field notes. This immersion in the data allowed them to identify patterns and meanings, and reflexive notes were generated to capture the researchers’ interactions with the data. This comprehensive understanding of the context facilitated the transition to the second phase, where initial codes were generated using Atlas.ti software.

In the second phase, data points were labelled, ranging from descriptive to interpretative categories. This coding process laid the groundwork for the third phase, which focused on clustering these codes into overarching themes. These themes encapsulated higher levels of understanding and were directly relevant to the research questions [70]. The third phase served as a pivotal point, bridging the coding process with the subsequent thematic development. It was a crucial step in ensuring that the themes were aligned with the research objectives.

The fourth phase entailed a rigorous review of these themes. The researchers revisited the data and consulted with external expert researchers to ensure the themes’ validity and reliability. This validation process was crucial for the integrity of the research findings. It also involved re-reading all the data to confirm that the final themes captured the important findings of the set. In the fifth phase, each theme was meticulously defined and summarized. Quotes from the data set were incorporated, and connections were made to other themes and existing literature to provide a nuanced understanding. This phase deepened the analysis and carefully shaped the themes.

The sixth and final phase involved the logical organization and presentation of these themes in this report. This phase was instrumental in offering a coherent and comprehensive argument in response to the research questions. The themes were organized in a manner that provided a clear narrative and effectively communicated the research findings. The resulting themes and write-up aimed to present a feasible and coherent argument in response to the research questions.

To further ensure the credibility of the research, the researchers employed member-checking and triangulation methods. Multiple data collection methods, including interviews and observations, were used. During the triangulation process, participants reviewed all final quotes and illustrations to confirm their accuracy. This approach aligned with the recommendations of [72], who emphasized the importance of using multiple data collection methods across time. Employing these methods in various settings and at different times allowed the researchers to mitigate the limitations of using a single method, as noted by [73].

## Results

The results in this study were derived from an analysis of findings from 11 interviews of university teachers at one federal university who taught in the rotation modality—two weeks face-to-face and two weeks online—as they gradually returned to face-to-face teaching and learning after more than a year of complete remote learning. This section is organized based on the themes resulted from the thematic analysis of the data. Summaries of findings from all participants are included, along with quotes and details from individual participants that support each theme.

### 1. Continuously changing expectations

The first theme indicated that expectations for university teachers were fluid and changing during biweekly rotation learning, which made it difficult for teachers and students to meet expectations as originally outlined in the course syllabus. Most participants lamented their decreased performance during the shift from complete online teaching to the rotation modality, and this reality check came as an unexpected surprise. They felt the need to lower their expectations, especially when it came to student engagement and curriculum teaching. As for engagement, the university teachers employed a variety of tactics to keep their students engaged, such as assigning small groups for class discussion or dividing the class into different virtual rooms in online sessions for collaborative work assignments. However, it was unclear whether students were participating in these sessions as many of the students did not turn on their cameras or microphones. Particularly in undergraduate courses with large numbers of students, teachers Amna, Diana, and Saif were baffled that they could not determine which students had turned off their cameras or simply walked out of the room while remaining logged-in. Sami, another teacher who experienced this shocking behavior, elaborated on his experience:

*I thought the students will change their behavior after moving to this phase [biweekly rotation learning]* … *The face-to-face period might make an impact on their engagement and motivation toward learning in the week offline … and [students] might feel compelled to try harder in the online sessions*. *But no sir! They act the same as they were before in the remote learning… even though I am glad they are engaged in the face-to-face period. But the online is still an issue*. *My expectation got lowered*.

As a reality check, university teachers believed that shifting to biweekly rotation learning would motivate the students a little bit and keep them engaged in the online sessions, but this was not the case. However, the teachers were optimistic during face-to-face sessions because there was a feeling of constant engagement and interaction with their professors and other classmates. Unfortunately, this feature vanished once again at the next online session. Surprisingly, even those who engaged during face-to-face sessions tended to become silent and passive in online sessions. In one case, Yousef felt forced to lower expectations and go with the flow during the online sessions of biweekly rotation learning. He stated:

*I lowered my expectation*, *and I try to prepare that I’m going to teach online whether they attend or they don’t… I plan to teach virtual class*. *If anyone showed up it’s fine*, *but if no one showed up*, *it’s fine*. *In the first few days it was really difficult for me; I really wanted them to attend those online sessions*, *but almost nobody attended*. *So what do I do*? *So I tried to lower my expectations and go with the flow*.

The situation became even worse when Yousef attempted to upload recorded videos of the lectures in hopes that students might watch them in their free time and reduce the tension for attending at the scheduled time of the class. He noted that no one viewed the recorded sessions. He continued:

*I have friendly conversation[s] before we start classes*, *and I ask [the students]*, *why didn’t you watch the recorded lectures*? *They say*, *we don’t like recorded lectures; we want you to talk*. *Then I said OK*. *So after that*, *I tried not [to] use recorded lectures*. *That’s one of the issues that I had*.

As far as the curriculum, university teachers lowered their expectations as they taught some curriculum topics. As detailed earlier, the new system ran two weeks face-to-face and two weeks online, but by no means was it purposefully structured as blended learning in spite of looking like it. Many university teachers scrambled to shift and distribute curriculum topics within their courses to align them with the teaching environment that would be used for them––whether face-to-face or online. Teachers who had practical courses found it difficult to adapt to online weeks when their labs and other practical lessons were longer or could not be altered to fit the rotation modality. This resulted in lowered expectations for curriculum completion, project submission, exams, and even attendance. Reem expounded on her decision to lower expectations when teaching under such circumstances:

*It was too much [of a] burden to teach practical topics in online period*. *How can you teach science and experience [a] different scientific phenomenon that is supposed to be taught in labs with different equipment and tools that are not in online*! *Virtual learning does exist*, *but [it is] not as effective as when learners use all their senses in a real learning experiment context*.

Other university teachers in majors like medicine, engineering, and applied education shared similar experiences. Michel noted that for some courses, he could create the right balance, but the situation became disturbing when the practical content was longer or more dominant than the theoretical portions. Amna and Anwar structured their priorities in organizing their courses by making compromises here and there as not all course content could be evenly distributed within the biweekly rotation model. This led them to reduce their expectations of being able to cover all the curriculum, a sentiment that echoed throughout the interviews with participating teachers.

Throughout their observations during rotation learning, the researchers noted that the university teachers repeatedly made vast reductions in their teaching expectations, especially during online weeks. Nonetheless, when comparing student learning during complete remote learning to biweekly rotation learning, students made noticeable progress in the latter. However, any progress was a result of the face-to-face class sessions, not the online sessions. Similarly, teachers and undergraduate students seemed more comfortable teaching and learning practical content, but only during the face-to-face portion. University teachers tried to enthuse students to engage in online sessions, but in most cases, students kept quiet and passive with cameras off and microphones muted. While non-participation was the norm in online sessions, there were some exceptions. In some few classes, participation rose when a few outgoing students engaged with the instructor, which encouraged more passive students to follow suit.

### 2. Mixed feelings regarding technology self-efficacy

The findings indicated that the majority of participating university teachers had never trained or engaged in professional development sessions centered around utilizing technology in either online or blended learning to keep them up to date with best technologies and applications. The vast majority of teachers mentioned that using Blackboard Ultra made the March-2020 transition to fully online learning easier, while the adoption of the rotation modality in 2021 created many difficulties for university teachers. The lack of training during the transition only added to those difficulties. For example, Diana noted:

*…Absolutely no training at all*. *I’ve never*, *ever received any training*, *and when I came to the university*, *and I started during the blended learning approach*, *which was a year ago*, *nobody taught me anything*. *I had no instruction*. *I knew it was coming*, *and I started doing a course with Cambridge university on online teaching*, *but to be honest*, *it didn’t really help me much*. *What I needed was real training on using the technology––not the how to teach*, *but the technology*, *the hardware*.

A few teachers mentioned having training from the university for using Blackboard Ultra, Microsoft Teams, and Zoom during the fully online phase. They stated that such trainings “set the tone for the course” in showing them how to “use different features” that seemed effective when used in online sessions. For the majority of teachers, however, the rotation modality was challenging and required more effort than the fully online learning just before the transition away from it as COVID-19 began to subside. They had mixed feelings about their ability to implement technology in this new emergent situation of biweekly rotation learning.

The participants premised their claims on the notion that the currently implemented rotation modality was not the same as blended learning. They asserted that it warranted focused training for technology use and teaching strategies in order to redeem its effectiveness. Michel stressed that this was only a phase-in stage before eventually moving back to complete face-to-face learning. He clarified the difference between the rotation modality of learning and blended learning:

*Well*, *we didn’t actually … [go] to blended learning*. *The two weeks online and the two face-to-face shouldn’t be considered as blended learning*. *It was more like an emergency teaching-learning situation*. *It looks like blended*, *but it is not*. *Blended learning is a different method*, *a different way*. *You … [use] platforms*, *and the intentions and the goals*, *or the objectives*, *of blended learning …[are] different than the two weeks on*, *two weeks off*! *It is an emergency [situation] before we get to full face-to-face [learning]*.

Along the same lines, Hessa shared her thoughts on the matter:

*I wouldn’t really call it blended learning*. *I guess that part of it was in the [classroom] and part of it was at home*, *but it wasn’t purposefully structured*. *It wasn’t like we … [were] looking at the course and we decided that these aspects of the course … [were] better to be taught online*, *and these aspects of the course … [were] better to be taught face-to-face*. *That would be for me a true blended learning approach; what we had was two weeks on and two weeks off*.

Upholding the commitment to use effective technology during the two weeks of online learning while keeping up with the two weeks of face-to-face learning emerged as the central challenge for university teachers. As Lexi echoed during her interview, “What you use in face-to-face is different from online sessions,” and each requires “different application use in technology.” All participants admitted that the teaching and learning process during the face-to-face encounter was more “meaningful,” “enjoyable,” “fruitful,” “educational,” “communicative,” and “observable.” However, the university teachers sought to find the right technology to compensate for the lesser results seen in the two weeks of online learning. Many acknowledged they actually “learn[ed] by doing” and that they could “play around” with different platforms and programs to figure them out. In grappling with the use of new technology, Amna narrated her experience:

*Online teaching improves my ability to use technology and gave me some time to learn*. *At first*, *I … struggled a bit*, *and during this new system [rotation modality]*, *it is more demanding because you need to see results*, *not only in face-to-face*, *but more importantly*, *in online sessions*. *With self-practicing and exploring different software and applications*, *I think that I’m good*, *and I’m much better now with technology*. *My ability improved a lot*.

In similar fashion, veteran university teachers found it more challenging to adapt to the new rotation modality of teaching and learning. Even so, they were up to the task to do whatever it took to see observable results. Yousef added that “learning technology might be easier if we get trained on [a] certain set of plausible technological applications…, but this situation is understandable since every course is different, and learning outcomes and objectives [are] different, which makes the whole situation cumbersome. Moreover, Maha noted that there was a “learning curve involved in the use of technology and being able to get a handle on using it properly.” All teachers unanimously agreed on one statement: “If [the] college offers professional development in the use of technology during the online weeks to bridge the gap in the learning process, then it would be … the most efficient and effective way to leverage technology to support teacher learning.”

On the other side of the experience spectrum, the newly joined university teachers felt relatively comfortable clicking around until they figured things out; however, “not all generations are not comfortable with that.” Sami reverberated this notion when he stated, “I am a technology instructor, so basically [a] big part of my job is to keep myself abreast and renewed about technology and effective applications for teaching and learning.” Another teacher, Anwar, had studied different online platforms about technology and teaching that simulate online and blended learning in his previous degree, which made him feel content about the situation. Similarly, Lex felt ready for the new system and was “very thankful” for the great experiences she had with training using different platforms and applications during her Ph.D. practicum before she joined the university.

During their observations, the researchers noted that during the rotation modality, the university teachers seemed to employ a variety of applications, especially in the online sessions, with the hope of engaging students and making learning more fun and enjoyable. Additionally, university teachers employed fewer technological tools during the face-to-face classrooms and seemed to feel less pressure keeping students engaged as the classroom was fully observable and in the open, unlike the virtual classroom where things were concealable. Despite the fact that all university teachers attempted to integrate various technological tools into their online sessions, some seem to have an extra measure of confidence in doing so. However, those who struggled had to waste a significant amount of valuable time figuring out new software and platforms. Furthermore, the observed self-efficacy of teachers indicated a significant gap between those with prior experience with new technology and those with little or no experience.

All in all, university teachers experimented with a variety of technological tools to engage students during online learning sessions. There were mixed feelings regarding technology self-efficacy depending on prior experience: some felt comfortable employing technology while others felt they struggled a bit. The rollout of the new rotation modality, which followed a year-plus stint of complete remote learning, was met with much difficulty, with very little or no professional development offered at the university, college, or even departmental levels. This left university teachers on their own to figure out how to battle with technology in their classrooms.

### 3. Rearranging the curriculum plan delivery to avoid learning loss

During the first couple of months after the implementation of the rotation modality of learning, university teachers were asked to continue their current curriculum into two different learning modes: online and face-to-face. Teachers found it challenging to prioritize content in a way that fit the best interests of students. Even more challenging, as echoed by many university teachers through interviews, was the struggle to compensate for the noticeable loss of learning on the part of students during the online sessions. At times, teachers had to repeat some of the topics during the face-to-face lessons to ensure learning was happening. Amna voiced her thoughts on repeating topics in-person that originally took place in online sessions:

*When [we] come together to face-to-face and [I] shower my students with questions about what we learned in previous weeks of online…*, *I am devastated to see how learning loss happens… What … [were they] doing during the online sessions*? *Sometimes I even wonder*, *were they even listening*? *I decided to use [a] portion of the face-to-face time to compensate for the learning loss of several important topics*

University teachers Anwar, Diana, Reem, and Sami expressed their frustration toward online learning as many students did not tell them when they did not understand the material being covered. Rather, they were prone to acting out, remained silent, or hid, and online sessions were hard to control. Reem reflected on her experience and shared some reasons why teaching her content was a bit harder in online sessions than in face-to-face sessions:

*I’m teaching to a black screen… I cannot see the students*. *Nobody is interacting with me*. *I don’t know what they’re doing*. *I have no idea what the students are doing*, *and there … [is] nothing I can do about it*. *There is nothing you can do*, *so I couldn’t overcome that challenge*. *It was a challenge*, *and I couldn’t do anything about it*, *and I don’t know many teachers that could because nobody can even see the students*. *What can I do*? *I mean I can’t go to their house and make sure that they’re interacting*. *There was nothing I felt I … [could] do to force them tell me if they understand the materials or to engage in class*.

Maha and Hessa felt a strong sense of urgency to make up for what was lost during biweekly rotation learning. They began to rearrange their course curriculum by scheduling lessons that seemed more passive for days when class was held online, saving lessons that seemed more active for face-to-face weeks. Despite the fact that the proportions were not equal and this technique of dividing was not very thorough, it seemed to be a necessary response to counter the negative effects of the online weeks and minimize learning loss as seen in decreased engagement, decreased understanding, and decreased participation. Amna’s courses were arranged to maximize student learning under the rotation modality of learning. She explained:

*Students are not missing out … [on teaching] in online periods; rather*, *their learning somehow is lost in the process… Learning*, *for example*, *cannot happen in a vacuum*. *There should be interacting*, *reflecting*, *asking questions*, *sharing*, *and more for learning to happen*. *It is not instructor centered*. *With all these reasons*, *I took some of the face-to-face weeks to make up for the learning loss*.

Saif shared a similar experience and shared that he prepared for his lessons, brought resources, and set the environment to maintain a climate conducive to learning both online and in-person. He remained reachable and available to all students, but there was nothing he could do for those who were not interested in learning. He clarified his reasoning:

*I did an activity today in class*, *and I went around*, *and I gave them papers and pencils*. *So the students sat there*, *and they didn’t do anything*, *and I go around and tried to encourage them*, *and I asked them*, *and they feel pushed and do it*. *So*, *if those students were at home and I can’t see them*, *they’ll never do the work*. *They’ll do it eventually because I’m pushing them*, *but you can’t make people do something if they don’t want to do it*, *and if nobody is watching them*, *I feel that the majority [of] students did not do the activity*…

Looking at the issue from a different perspective, some university teachers felt the students were responsible for their own learning and should take ownership of it. While they strongly agreed that the online sessions were a setback and kept students behind, they felt that students should take control of their lives, accept responsibility, make good decisions, and make changes as needed. They are not in grade school anymore. They are not kids anymore. They are adults! Reem echoed the sentiments of university teachers in stating that their responsibility was to create an opportunity for learning, and the rest was up to the students.

The observations conducted by the researchers corroborate university teachers’ accounts in most cases. For instance, it was apparent that many university teachers believed there was learning loss in the rotation modality, especially in the online sessions where passivity, less engagement, and less interaction prevailed. To compensate for the purported learning loss, many university teachers felt the need to revisit some topics during face-to-face sessions that had been previously taught in online sessions. Others attempted to divide the course syllabus to fit the nature of rotation learning while still looking out for the best interest of the students. Others yet delivered their course content just the way it was initially planned because they were ordered in logical sequence, and one topic was a prerequisite of the next. In such cases, changes to the curriculum were not the optimal solution, and they went with the flow of the implemented rotation modality.

### 4. Trial and error with teaching strategies

The collective message shared by university teachers across the board was their efforts to continually experiment with new teaching strategies in the biweekly rotation learning modality. For them, trial and error were one of the most effective forms of learning, and university teachers felt the need for this because most had no training on what effective teaching and instructional strategies were best suited to the emergent situation. Michel, for instance, stressed that university teachers who were forced to teach under this new environment had to depend too much on trial and error as a strategy to gain knowledge and figure out what produced better results. The following statement made by Saif supported that notion: “When we make an error––mistakes, you know!––or fail at something, it is an important moment because we give ourselves an opportunity to reflect and analyze that failure, and then make proper changes, then try again.” Some university teachers recognized that there were many teaching strategies on the horizon, but they wondered which was the most effective. Lexi expressed her experience:

*We have to be careful with blended learning … which course*, *which type of students and their learning styles*, *why use of this teaching strategy*, *is it good for the student or its just for doing the blended*? *I mean the two weeks offline and online … The idea is to help students learn*. *At the start*, *it was completely hard on them to be more independent … There was [sic] a lot of challenges*, *but as I said*, *by practice and improving my skills*, *I tried different instructional strategies [to] see what are most efficient*.

Trial-and-error teaching is a universal strategy for establishing which actions are beneficial or ineffective in a new environment. By enacting foundational strategies, university teachers can improve their teaching practices. In explaining his experience with the rotation modality, Yousef saw his classroom as an experiment lab where teaching strategies could be checked over and over. In addition, Sami approached one of his colleagues within the same department and discussed recent pedagogical strategies they had employed, their failures, and their successful instructional strategies. Sami stated:

*We are in a rush to finish our course curriculum … We feel pressured in several areas to try out things in [the] classroom because no training was initiated prior to the implementation of this so-called blended learning*. *I think … hmm … sharing what we are doing with our colleagues is one of the best ways to accelerate the process of learning … and disseminating our teaching failures and successes*.

As illustrated in his statement, Sami believed that university teachers could help their colleagues grow and improve by sharing their trail-and-error cycles of instructional practices during the implementation of rotation learning. Furthermore, changes in instructional practices may come as a response to student learning. Amna, for example, emphasized the changes she made to her practice to prevent her students from getting bored: “The main issue is don’t guide yourself to feel boring … because any strategy, after a certain time, it will be boring for students.”

The observation rounds in in-person classrooms and online sessions showed that not all teachers were persistent in continually adapting their instructional practices. It is also worth mentioning that teachers seemed to try things out in online sessions more than in face-to-face classrooms. Some of the teaching strategies employed in the rotation modality included flipped classrooms, digital learning, problem solving, active learning, and case-based learning. University teachers tended to be more open to changes at the beginning of the rotation modality, but this trial and error of instructional practices began to fade at the midpoint of the semester when midterm exams began.

### 5. The need to consult with students in the teaching and learning process

University teachers revealed that one of the key methods of assistance in adapting and transforming the teaching and learning process was consultation with their students, especially in times of crisis. This theme was reverberated by most of the participants, who saw their students as valuable assets as they were involved in the decision-making process to enhance teaching and learning during the implementation of the biweekly rotation modality of learning. Anwar and Lexi admitted that teachers in the rotation modality operated in a whirlwind environment and that students’ input could be of help with finding the best-fit teaching strategies, materials used, exams, etc. Diana further clarified why it was necessary to include students in the teaching and learning process in the rotation modality:

*I think what we are experiencing is shocking everyone … and under these circumstances*, *where you cannot control everything*, *it is better to look from different perspectives*, *by which I mean students’ [perspectives] … Because as a teacher*, *you may have some cues and hints in face-to-face [classroom] that tells [sic] you if everything is going well or not in class*, *but this is not something you can see in online sessions*. *So*, *consulting with students about certain aspect[s] like technology or teaching methods can be a good strategy to understand what and how they [students] feel about [them] … you know*, *to make them more engaged and active …*, *[which] bring[s] good results*.

In similar vein, Michel and Maha shared their perspectives and noted that engaging students and consulting them about different aspects of the classroom with regard to teaching and learning was important at this critical time in education. Moreover, allowing students to give their input and thoughts on what is implemented in the rotation modality can provide a clearer picture about their performance. Another teacher participant in this study, Saif, stated the following: “In my case, this benefits everyone … Students feel more engaged in their learning, and I feel more adaptable to make changes to what they believe, if this will bring prosperity to [the] classroom.” Multiple other participants also shared examples of times in which they changed their classroom planning and agenda according to what their students suggested. For instance, Saif made some changes in group assignments in his class to increase discussion and interaction, and Diana used Blackboard Ultra to meet with her students instead of Zoom and Microsoft Teams for better access and options. Yousef recounted a number of incidents in which he directly invited his students to evaluate applications, teaching methods, ways of communicating, and more to get them onboard and engage them in the learning process. He explained:

*As I told you earlier*, *I recorded some lectures for my students*, *but no one listened to them*. *And I asked them why they said we prefer interacting and asking the instructors directly … They think recorded videos are boring to listen to … They told me they want interactive PPT slides to make them engaged … [and] asked to have more time to think when I asked questions*.

For Yousef and other participants, giving students a voice in the classroom fostered a stronger learning environment and accelerated the refinement of teaching practices to better align with the needs of the emergency rotation modality of learning. Maha added that waiting and reading student evaluations at the end of the semester was too late to make the required changes in the rotation modality. She saw seeking student input as an urgent practice to be able to respond to students’ needs and have a clear direction for improvement during self-reflection of their teaching as well as assessment of learning throughout the semester. This was particularly true given the many daunting factors in the rotation modality, such as no training, no knowledge of applications and technology, cameras being off during online sessions, students absence, and student silence. Consultation with students was an open invitation for improving instructional practice by gleaning insights from multiple perspectives––whether in the form of student praise or criticism––to arrive at a clearer understanding of way to improve.

During the observation rounds of both online and face-to-face classroom sessions, it was clear that there were a few direct invitations from university teachers asking about certain issues, such as what platforms to use for online sessions (Microsoft Teams, Zoom, Blackboard Ultra), what applications students preferred to use to share their responses during class, and how assignments should be approached (i.e., in groups or individually). In these incidents, students either spoke directly about what they preferred or decided based on poll results. It goes without saying that these invitations occurred during the online sessions. However, there may have been some invitations for student input during office hours, email exchanges, or anywhere outside the classroom, which the observations would not have been able to capture.

### 6. The shift from struggle to resilience

As the research participants shared their challenges and changing self-efficacy in various aspects of the biweekly rotation modality of learning, resilience revealed itself as a repeating element. All ten participants included some reference to resilience, strength, or pride in the face of the emergency remote teaching throughout the semester. University teachers assumed the rotation modality would have been easier for them after a year of remote learning, but that was not the case. The lack of technology efficacy and lack of preparation for how to accommodate the alternating two weeks of face-to-face learning and two weeks online pushed proficiency and confidence further out of reach. All university teachers voiced that there were struggles, frustrations, and a lack of self-confidence in their teaching abilities at some point during the implementation of the rotation modality. Saif elaborated:

*It is frustrating at the beginning because you feel you know face-to-face*, *and you have been doing online sessions last year … But when they are combined*, *it is totally*, *completely different from what you expected and experienced*. *You want to do better at both*. *You see incomparable results*, *and you badly want to do better at both*. *This is a challenge in many ways*.

University teachers’ responses during interviews highlighted the challenges and struggles they faced, but also revealed their eagerness to keep moving forward and try different methods of instruction regardless of their unpreparedness or not being accustomed to the rotation modality. Amna, for instance, acknowledged struggling in some areas and pinpointed age as a potential factor. She stated, “Not being born within the digital generation, but playing around with technology would suffice to do the work.” She strove to add “technologies [that] appear[ed] to have value and enhancement” to her teaching. Many participant accounts typified the classroom, especially the online classroom, as an experiment lab in which university teachers could test out technology, applications, teaching strategies, and learning assessments to improve the teaching and learning process.

In most cases, university teachers made use of a trial-and-error strategy to cope with challenges and uncertainties in the wake of the new rotation learning modality. For example, Maha asserted that if a particular strategy was not effective, she would try something else. Anwar added that Google was a good place to spot new things and read reviews about technology and teaching strategies. Other teachers shared their experiences with their colleagues in a mutual effort to improve upon their practices, and sharing practices and collaborating was helpful in learning new content online, troubleshooting technology, and caring for the social and emotional needs of others. For instance, Lexi collaborated with other teachers who had her students in a different class in order to expedite the process of finding the best strategies for a particular group. Additionally, in order to minimize the burden of the various challenges, all university teachers agreed that consultation with students helped them make it through the semester.

Another notion that most participating teachers agreed on was that the unprecedented experiences they encountered during the implementation of the rotation modality would be useful in the future, such as in a recurring circumstance wherein they might feel the need to employ what they had learned. As a matter of fact, university teachers felt a sense of pride in their ability not only to survive, but also thrive, in making it through a difficult semester teaching in an emergency situation. Michel expressed his renewed pride and gains from the process:

*Regardless of the exhausting and puzzling semester that whoops on us with a surprise to do the rotation modality …*, *I felt like I achieved a lot and challenged myself no matter the lack of preparations and training … I tried*, *changed*, *adopt[ed] so many things in the way of teaching to get through to undergraduate students*, *and I am proud of myself for being able to do all of that*. *I feel like I was not only … [in a] survival situation*, *but actually the opposite––you know*, *kind of [a] thriv[ing] state*.

Most university teachers were satisfied with their accomplishments and felt pride in pulling themselves through the semester despite being overwhelmed with the various challenges of the rotation modality. While they had a sense of being underprepared, this new experience made them aware of their abilities and potential.

Throughout the online and in-person classroom observations, the researchers noted the difficulties that university teachers faced, particularly at the beginning of the rotation modality in the online sessions. In some cases, the anxiety and confusion were visible during the first weeks of implementation. Then, teachers gradually became accustomed to the new experience. Finally, they adopted an attitude of thriving and pushing forward by trying out new teaching strategies, playing with different technologies, and rearranging their curriculum to better counter learning losses and other learning deficiencies. Although their actions and behaviors portrayed struggling in their classes with undergraduate students, they demonstrated resilience in their ability to face and overcome challenges.

## Discussion

Each theme outlined in the results section was further analyzed and interpreted in terms of participants’ perceptions––whether they supported or countered the literature––and through the lens of self-efficacy. The six themes were interpreted separately with rich information infused by participants’ first-hand experiences to create a deep view of the analyzed data. Furthermore, the perceptions of ten university teachers at one federal university along with current literature on teaching during the pandemic crisis were used to interpret self-efficacy.

### 1. Continuously changing expectations

The first theme that emerged from the collected data was the changing expectations of university teachers and students during the new biweekly rotation modality of teaching and learning—two weeks face-to-face followed by two weeks online. These findings coincide with the findings from the literature, which showed that as the pandemic was a fluid and changing emergency, expectations of teachers and students who were going through that emergency also changed [74]. The participants elucidated that they found it challenging to engage students, particularly in online sessions, in spite of enormous efforts to employ tactics and a variety of technology applications. [75] noted that the emotional and physical presence of students at their computers was difficult to ascertain in a remote learning environment. In addition to student engagement challenges, university teachers lowered their expectations in many areas, including curriculum, assignments, projects, and attendance. For them, their malleability as teachers was paramount to the success of learning during the emergency teaching situation. [76] found that teachers’ self-efficacy during the pandemic may have been influenced by their ability to adapt to constant changes. While most of the university teachers in the study felt some measure of success, they cited challenges related to their ability to adapt to changes, such as lowering their expectations for students and course requirements in the rotation mobility of teaching in the COVID-19 pandemic.

### 2. Mixed feelings regarding technology self-efficacy

Most university teachers in the study had no prior training or professional development on using technology as the rotation modality took over complete remote learning as part of the gradual shift back to the face-to-face classroom. They spoke of the urgent need for their university to be responsive to the emergency situation and provide teachers with professional development to nurture their technology skills, use of innovative and effective instruction applications, and showcase cutting-edge practices to meet new demands. [77] stated that teachers were unprepared for emergency remote teaching from a lack of prior professional development or in-class teaching experience. However, even with the absence of professional development, all participants were determined to exercise a trial-and-error strategy to master new technology, and they believed that effective use of technology required refinement and continual improvement. Nonetheless, not all teachers expressed the same level of efficacy, where some felt comfortable playing around with various technology applications and others struggled in the learning and implementation process, especially those with no experience. [32] concluded that educators who had experience with online teaching before the pandemic reported high levels of self-efficacy, and those with little or no prior experience reported having difficulty teaching. The findings related to the gap in technology efficacy among teacher participants at the university were, therefore, consistent with current research. Efficacy can be further explained in this way: “The extent to which teachers perceive such efficacy may influence whether or not they take action, invest effort in an action, and how long they may sustain possible challenges” [78]. As a result of teaching unfamiliar curriculum on a previously unused learning platform, university teachers likely saw a shift in their self-efficacy. [79] supported this notion when he stated, “The COVID-19 pandemic has created a situation in which usually efficacious teachers may not feel efficacious now” (p. 3).

### 3. Rearranging the curriculum plan delivery to avoid learning loss

Most teachers were concerned by the loss of learning that resulted from the emergent rotation modality, which forced them to adapt their course content to fit what was best for students. Emergency remote teaching can often push teachers to reinvent the way they employ content [36, 80, 81]. Unsurprisingly, as the rotation modality is an emergent situation, university teachers stressed the enormous amount of time they needed to reconsider and rearrange the content of their courses. This finding aligns with [80] research, which found that planning for curriculum and teaching was a burden of time commitment that seemed to multiply in the face of remote and hybrid learning. Moreover, multiple university teachers indicated that learning at higher education institutions urged undergraduate students to develop a sense of responsibility toward learning and take ownership of it, especially when the opportunity and environment were already established for them. Even after doing all they could to arrange their course curriculum to fit the rotation modality, the teachers recognized that in the end, students had the final say in whether they participated and learned the content.

### 4. Trial and error with teaching strategies

In theme four, most of the university teachers described the rotation modality as an experiment arena for trial and error with instructional practices aimed at enhancing student learning. This trial-and-error strategy was characterized by repeated and varied attempts at finding a teaching strategy or activity that best suited the unfamiliar environment of the rotation learning modality. Although there were some differences in the observed use of trial and error in face-to-face and online sessions versus what participants stated in interviews, it is undoubtful that such ongoing changes and experimenting improved instructional practices. This finding goes hand-in-hand with what [82] found, that continually experimenting and learning from trial and error can set educators up for online teaching success if they view this new style of teaching as a growth experience. [83] further noted that the era of emergency remote teaching created “the need to invest in different modes of instructional designs… to ensure proactive movement in instructional innovations and teachers’ training and development” (p. 150).

### 5. The need to consult with students in the teaching and learning process

In theme five, university teachers underscored the need for student involvement in teachers’ decision-making process during the rotation modality of learning in order to arrive at a clearer understanding of classroom successes and challenges and better shape the classroom experience. This is particularly true as the students are the recipient of teaching and can give teachers clues about many aspects of educational initiatives that might help them gain a better sense of what students prefer to motivate and boost their learning. In his study, [84] accentuated the significance of student input to open the door for educators to understand the teaching context, make better sense of their doings, and see the direct impact of implemented models in the learning process.

### 6. The shift from struggle to resilience

In theme six, university teachers felt they grew in perseverance as a result of their struggles during the 2020–2022 complete remote learning and biweekly rotation modalities. Challenges arose in different areas, such as in technology, curriculum, and teaching and learning; yet, despite the imposing pressure upon them, the teachers felt motivated to adapt, change, and succeed. They expressed a sense of pride in their achievements as a result of the experience. [28] highlighted self-efficacy as a result of internal persistence and effort that resulted in the motivation to flourish and obtain desired outcomes. The university teachers interviewed in this study had some positive self-efficacy from the outset, and their accumulating experiences compounded by motivation and a strong desire to succeed over time gave birth to a strong sense of perseverance. [36] noted that high self-efficacy resulted from perseverance, recognizing and meeting challenges, and making concerted efforts to achieve success. Additionally, when teachers perceive success, their perceived self-efficacy grows as a result of the mastery experience [79].

## Conclusion

The impacts of rotational blended learning on university teachers were both positive and negative as observed in this study. On the positive end, when the teacher participants had high self-efficacy, it manifested itself in all areas of their teaching practice, including the use of technology, curriculum delivery, and implementation of new teaching strategies. However, the introduction of the rotational blended learning caused a significant decrease in their self-efficacy, which left the teachers feeling unconfident in their jobs. In order to effectively combat and minimize the decline in self-efficacy, administrators, heads of departments, and deans must create a collaborative, experiential, and supportive educational environment to fulfill the requirement of total quality management [85]. This study has examined the changing self-efficacy of teachers as they struggled with teaching students at the undergraduate level due to the implementation of new technologies, different curricula, and new methods of teaching in the wake of the COVID-19 pandemic.

The change to two weeks of teaching online followed by two weeks in person came as a surprise to educators, and that surprise recurred with the return to fully in-person education just as they were beginning to become accustomed to the rotational method. In order to properly observe and record the impacts of these changes on teachers, this qualitative case study employed many observations and face-to-face, semi-structured interviews with 11 university teachers teaching undergraduate students. The findings, which derived from a thematic analysis of the data, revealed six themes regarding how teachers viewed their experiences with rotational learning: (1) continuously changing expectations; (2) mixed feelings regarding technology self-efficacy; (3) rearranging the curriculum plan delivery to avoid learning loss; (4) trial and error with teaching strategies; (5) the need to consult with students in the teaching and learning process; (6) the shift from struggle to resilience.

This study has several implications for improved professional practice and additional research. It uncovers the challenges created for university teachers of undergraduate students upon implementation of the rotational model of blended learning in response to the COVID-19 pandemic. In future cases where emergency remote teaching is required, it would be wise to train the teachers on the most effective teaching methods to use in hybrid or fully-online learning environments. Furthermore, school administrators should train their teachers sufficiently in the use of new and best-suited technologies to assist them in their virtual classrooms. Participants consistently noted an increase in self-efficacy as they became better equipped with the use of technology and as they learned new methods of teaching that were better suited for the online environment. It should be noted that in emergency situations, changes and actions should be measured carefully and implemented in an orderly way with sufficient guidance for all shareholders to be able to proceed without chaos and confusion. Higher education institutions need to create an environment that is supportive, collaborative, experiential, and conducive to developing the teaching practices of university teachers. Such an environment allows teachers to grow in self-efficacy, which in turn strengthens their teaching performance in all areas.

This study provides university teachers with multiple experiences and solutions from other teachers. Theme six, in particular, demonstrates that with enough guidance, training, and collaboration, teachers can reach a point of resilience and confidence. Trial and error may also serve as an impetus for improved teaching practices, especially when those trial-and-error experiences are shared with colleagues. By collaborating with other teachers on what has worked in a hybrid environment and things to avoid, the path toward resilience can be shortened significantly. It is also important to note that there is no risk or harm in simply asking students themselves whether certain teaching strategies are effective for them.

It is not a surprise that the efforts of university teachers, both individually and as groups, directly affect their self-efficacy. For that reason, administrators within universities must create environments that encourage collaboration among teachers. University administrators should also focus on self-efficacy with more energy in order to directly better teachers in that regard. Some factors that contribute to teachers’ self-efficacy include thorough training in various technology applications, conscious awareness of the need to adapt the curriculum to new teaching modalities and practice with pedagogical strategies that help students engage more actively in the online setting. Future research might include a mixed-method study that combines the benefits of quantitative and qualitative data collection methods and uses a larger sample pool in order to more thoroughly understand the challenges teachers face with rotational learning. Additionally, a comparative study might discover proven best practices of teaching for rotational learning at federal and private universities across the United Arab Emirates. Finally, while this study demonstrates that mastery of practice and understanding of technology are contributors to teachers’ self-efficacy, future research might focus on how educational institutions can implement programs to build up their teachers’ self-efficacy.
